# Editorial: Beyond chemotherapy and immunotherapy in thoracic malignancies: Overcoming resistance by tackling new molecular pathways

**DOI:** 10.3389/fonc.2022.997404

**Published:** 2022-09-16

**Authors:** Giannis Mountzios, Giuseppe Luigi Banna, Christian Rolfo

**Affiliations:** ^1^ Fourth Oncology Department and Clinical Trials Unit, Henry Dunant Hospital Center, Athens, Greece; ^2^ Portsmouth Hospitals University, National Health System (NHS) Trust, Portsmouth, United Kingdom; ^3^ Center for Thoracic Oncology, The Tisch Cancer Institute, Icahn School of Medicine at Mount Sinai, New York, NY, United States

**Keywords:** thoracic malignancies, lung cancer, chemotherapy, immunotherapy, resistance, molecular pathways

Chemotherapy and immunotherapy, administered sequentially or concurrently, represent the current standard of care for most patients with advanced non-small-cell lung cancer (NSCLC) and other thoracic malignancies (1). However, even when administered as a combination in first-line treatment of NSCLC, chemoimmunotherapy confers a median progression-free survival (PFS) of approximately 8-10 months and a median overall survival that falls below two years in both squamous and nonsquamous histologies (2–6). Immune checkpoint inhibitors (ICIs) administered as monotherapy in selected patients with high programmed-death ligand 1 (PD-L1) expression achieve better outcomes that, nevertheless, confer long-term responses only in a minority of patients (7–9).

Patients with advanced NSCLC who progress after chemoimmunotherapy, given either concurrently or sequentially, currently have limited treatment options. Second-line chemotherapy with docetaxel, administered alone or in combination with anti-angiogenic agents (ramucirumab, nindetanib), offers modest clinical outcomes (10, 11). ICIs given as monotherapy have been proven superior to docetaxel in patients progressing after first-line chemotherapy (12–15). Recent advances in elucidating the molecular biology of the disease have led to the identification of molecular pathways that could be exploited therapeutically in an effort to improve outcomes by overcoming resistance to immunotherapy and chemotherapy (1). The patterns of resistance to immunotherapy have been extensively studied and may involve primary or secondary resistance as well as intrinsic and extrinsic mechanisms encompassing diverse molecular pathways (16). Consequently, potential strategies to overcome acquired resistance may include, among others:

-Combinations of immune checkpoint-modifying agents that either inhibit suppression of immune cells (antagonists) or activate the immune system (agonists). Examples include the synergistic activity of PD-(L) 1 inhibitors with anti-CTLA4 or anti-TIGIT monoclonal antibodies, as well as the combination with agonists of immune activation, such as OX-40 monoclonal antibodies (17)

- Combinations of ICIs with molecular targeted agents, such as tyrosine kinase inhibitors, that interfere with diverse molecular pathways, including cellular proliferation, apoptosis regulation, inflammation, DNA repair, epigenetic modification and angiogenesis. Metabolic pathways that are often associated with immune regulation involve the mitogen-activated protein kinase (MAPK), the phosphatidyl-inositol -3-kinase (PI3K) and the signaling transducer and activator of transcription (STAT) molecular pathways (18)

- Biotechnological advances have enabled the clinical development of novel molecules with unique properties, such as antibody-drug conjugates and bi-specific antibodies, that may act as “engagers” of the immune system to cancer cells, triggering thus the anti-tumor activity of the immune system against neoantigens (19, 20)

- Finally, the role of the tumor microenvironment and the identification of potential biomarkers of activity/resistance to novel treatment strategies is currently a field of active investigation to enhance anti-tumor immunity by studying the interaction of the tumor cells with the stroma and the surrounding host niche (21)

In the current Research Topic of *Frontiers*, we present eight (8) high-quality articles that evaluate the strategies above to tackle resistance to chemo-immunotherapy. Bie et al. comprehensively reviewed anti-PD-1/PD-L1 immunotherapy-related mechanisms, drug resistance-related mechanisms, and therapeutic efficacy-related predictive biomarkers. Zhang et al. studied the Effect of the Extracellular Superoxide Dismutase (SOD3) Gene in Lung Cancer, revealing its prognostic value, while Wu et al described a novel gene, *SPTBN2*, which is highly expressed in lung adenocarcinoma, positively correlates with poor prognosis, and can promote the proliferation, migration, and invasion of lung adenocarcinoma cells. Yuan et al. evaluated the DNA epigenetic methylation modification pattern of patients with lung adenocarcinoma, which could enhance our understanding of the features of tumor microenvironment characterization and may promote more favorable immunotherapy strategies for these patients. In a pivotal phase I/II study, Kim et al. showed the feasibility and preliminary signs of efficacy of the combination of Dasatinib and Osimertinib in TKI-naïve patients with advanced EGFR-mutant NSCLC, suggesting a synergistic effect for EGFR and SRC inhibition in this setting. In an exploratory study, Schneider et al. tested various Acute Phase Proteins as Early Predictors for Immunotherapy Response in Advanced NSCLC. They concluded that higher pre-therapeutic levels of haptoglobin and ceruloplasmin are independent predictors of a worse PFS. Suo et al. studied the effect of prior ant-angiogenic agents on the efficacy of anlotinib in patients with NSCLC and concluded that it might negatively affect the PFS. Finally, Frisone et al. summarized the definitions of resistance to immunotherapy and examined its underlying mechanisms and potential corresponding treatment strategies by focusing on recently published clinical trials and trials that are expected to deliver results soon.

Discovering ways and strategies to overcome resistance to immunotherapy, chemotherapy and targeted agents is a constantly evolving research field. In this ever-lasting scientific endeavor, the only road to successful outcomes passes through sound scientific ratiional, robust pre-clinical experimentation and well-designed and executed clinical trials. The algorithmic use of potent biomarkers, including liquid biopsies, will likely assist in identifying the subgroups of patients who will benefit the most from each experimental strategy. The collective goal of these strategies will be to implement individualized treatment approaches after chemotherapy and immunotherapy failure, based on the unique biological characteristics and mechanisms of primary or acquired resistance for each one of our patients. This personalized approach paves the way for precision oncology, which is the optimal strategy to improve the lives of our patients.

## Author contributions

All authors listed have made a substantial, direct, and intellectual contribution to the work and approved it for publication.

## Conflict of interest

GM: Advisory and consultation fees: MSD, AstraZeneca, BMS, Roche, Takeda, Novartis, Amgen, Sanofi, Pfizer, Janssen Travel and accommodation fees: AstraZeneca, BMS, MSD, Roche, Takeda, Novartis, Amgen, GSK PI in sponsored clinical trials: GSK, Novartis, Roche, BMS, MSD, AstraZeneca, Merck, Amgen, Sanofi, Mirati, Gilead sciences. GB Consulting Fees: AstraZeneca Honoraria: AstraZeneca, Astellas. CR Consulting Fees/Speakers’ Bureau: ARCHER, Inivata Novartis Boston Pharmaceuticals EMD Serono Astra Zeneca Roche Guardanthealth MSD Pfizer Mirati Eisai COR2ED Daiichi Sankyo Sanofi Genzyme- Regeneron Physicians Education Resource, LLC. Intellisphere, LLC CEA Bayer U.C. LLC General Dynamics Ownership (Stock, stock options): Novartis.

## Publisher’s note

All claims expressed in this article are solely those of the authors and do not necessarily represent those of their affiliated organizations, or those of the publisher, the editors and the reviewers. Any product that may be evaluated in this article, or claim that may be made by its manufacturer, is not guaranteed or endorsed by the publisher.

## References

[1] Remon J Passiglia F Ahn MJ Barlesi F Forde PM Garon EB. Immune checkpoint inhibitors in thoracic malignancies: Review of the existing evidence by an IASLC expert panel and recommendations. J Thorac Oncol (2020) 15(6):914–47. doi: 10.1016/j.jtho.2020.03.006 32179179

[2] Gandhi L Rodríguez-Abreu D Gadgeel S Felip E De Angelis F. Pembrolizumab plus chemotherapy in metastatic non-Small-Cell lung cancer. N Engl J Med (2018) 378(22):2078–92. doi: 10.1056/NEJMoa1801005 29658856

[3] Paz-Ares L Luft A Vicente D Tafreshi A Gümüş M Mazières J. Pembrolizumab plus chemotherapy for squamous non-Small-Cell lung cancer. N Engl J Med (2018) 379(21):2040–51. doi: 10.1056/NEJMoa1810865 30280635

[4] West H McCleod M Hussein M Morabito A Rittmeyer A Conter HJ. Atezolizumab in combination with carboplatin plus nab-paclitaxel chemotherapy compared with chemotherapy alone as first-line treatment for metastatic non-squamous non-small-cell lung cancer (IMpower130): A multicentre, randomised, open-label, phase 3 trial. Lancet Oncol (2019) 20(7):924–37. doi: 10.1016/S1470-2045(19)30167-6 31122901

[5] Socinski MA Jotte RM Cappuzzo F Orlandi F Stroyakovskiy D Nogami N. IMpower150 study group. atezolizumab for first-line treatment of metastatic nonsquamous NSCLC. N Engl J Med (2018) 378(24):2288–301. doi: 10.1056/NEJMoa1716948 29863955

[6] Hellmann MD Paz-Ares L Bernabe Caro R Zurawski B Kim SW Carcereny Costa E. Nivolumab plus ipilimumab in advanced non-Small-Cell lung cancer. N Engl J Med (2019) 381(21):2020–31. doi: 10.1056/NEJMoa1910231 31562796

[7] Reck M Rodríguez-Abreu D Robinson AG Hui R Csőszi T Fülöp A. Pembrolizumab versus chemotherapy for PD-L1-Positive non-Small-Cell lung cancer. N Engl J Med (2016) 375(19):1823–33. doi: 10.1056/NEJMoa1606774 27718847

[8] Herbst RS Giaccone G de Marinis F Reinmuth N Vergnenegre A Barrios CH. Atezolizumab for first-line treatment of PD-L1-Selected patients with NSCLC. N Engl J Med (2020) 383(14):1328–39. doi: 10.1056/NEJMoa1917346 32997907

[9] Sezer A Kilickap S Gumus M Bondarenko I Ozguroglu M Gogishvili M. Cemiplimab monotherapy for first-line treatment of advanced non-small-cell lung cancer with PD-L1 of at least 50%: A multicentre, open-label, global, phase 3, randomised, controlled trial. Lancet (2021) 397(10274):592–604. doi: 10.1016/S0140-6736(21)00228-2 33581821

[10] Garon EB Ciuleanu TE Arrieta O Prabhash K Syrigos KN Goksel T. Ramucirumab plus docetaxel versus placebo plus docetaxel for second-line treatment of stage IV non-small-cell lung cancer after disease progression on platinum-based therapy (REVEL): A multicentre, double-blind, randomised phase 3 trial. Lancet (2014) 384(9944):665–73. doi: 10.1016/S0140-6736(14)60845-X 24933332

[11] Herbst RS Sun Y Eberhardt WE Germonpré P Saijo N Zhou C. Vandetanib plus docetaxel versus docetaxel as second-line treatment for patients with advanced non-small-cell lung cancer (ZODIAC): A double-blind, randomised, phase 3 trial. Lancet Oncol (2010) 11(7):619–26. doi: 10.1016/S1470-2045(10)70132-7 PMC3225192 20570559

[12] Brahmer J Reckamp KL Baas P Crinò L Eberhardt WEE Poddubskaya E. Nivolumab versus docetaxel in advanced squamous-cell non-Small-Cell lung cancer. N Engl J Med (2015) 373(2):123–35. doi: 10.1056/NEJMoa1504627 PMC4681400 26028407

[13] Herbst RS Baas P Kim DW Felip E Pérez-Gracia JL Han JY. Pembrolizumab versus docetaxel for previously treated, PD-L1-positive, advanced non-small-cell lung cancer (KEYNOTE-010): A randomised controlled trial. Lancet (2016) 387(10027):1540–50. doi: 10.1016/S0140-6736(15)01281-7 26712084

[14] Borghaei H Paz-Ares L Horn L Spigel DR Steins M Ready NE. Nivolumab versus docetaxel in advanced nonsquamous non-Small-Cell lung cancer. N Engl J Med (2015) 373(17):1627–39. doi: 10.1056/NEJMoa1507643 PMC5705936 26412456

[15] Rittmeyer A Barlesi F Waterkamp D Park K Ciardiello F von Pawel J. Atezolizumab versus docetaxel in patients with previously treated non-small-cell lung cancer (OAK): A phase 3, open-label, multicentre randomised controlled trial. Lancet (2017) 389(10066):255–65. doi: 10.1016/S0140-6736(16)32517-X PMC6886121 27979383

[16] Schoenfeld AJ Hellmann MD. Acquired resistance to immune checkpoint inhibitors. Cancer Cell (2020) 37(4):443–55. doi: 10.1016/j.ccell.2020.03.017 PMC7182070 32289269

[17] Rolfo C Ordóñez-Reyes C Cardona AF. Immunotherapy resistance in non-small cell lung cancer: A rubik’s cube to assemble. J Immunother Precis Oncol (2021) 4(4):185–8. doi: 10.36401/JIPO-21-7 PMC9138479 35665026

[18] Attili I Tarantino P Passaro A Stati V Curigliano G de Marinis F. Strategies to overcome resistance to immune checkpoint blockade in lung cancer. Lung Cancer (2021) 154:151–60. doi: 10.1016/j.lungcan.2021.02.035 33684660

[19] Desai A Abdayem P Adjei AA Planchard D. Antibody-drug conjugates: A promising novel therapeutic approach in lung cancer. Lung Cancer (2022) 163:96–106. doi: 10.1016/j.lungcan.2021.12.002 34942494

[20] Elshiaty M Schindler H Christopoulos P. Principles and current clinical landscape of multispecific antibodies against cancer. Int J Mol Sci (2021) 22(11):5632. doi: 10.3390/ijms22115632 34073188 PMC8198225

[21] Genova C Dellepiane C Carrega P Sommariva S Ferlazzo G Pronzato P. Therapeutic implications of tumor microenvironment in lung cancer: Focus on immune checkpoint blockade. Front Immunol (2022) 12:799455. doi: 10.3389/fimmu.2021.799455 35069581 PMC8777268

